# Clinical and electromyographic evaluation of botulinum toxin type A in the treatment of gummy smile: A prospective clinical study

**DOI:** 10.34172/joddd.2021.021

**Published:** 2021-05-05

**Authors:** Payal Padmakar Mate, Kumar Nilesh, Anand Joshi, Arun Panda

**Affiliations:** ^1^Department of Oral and Maxillofacial Surgery, School of Dental Sciences, Krishna Institute of Medical Sciences, Maharashtra, India; ^2^Department of Oral and Maxillofacial Surgery, Saraswati-Dhanwantari Dental College and Hospital, Maharashtra, India

**Keywords:** Botulinum toxin, Electromyography, Gummy smile

## Abstract

**Background.** The present study aimed to assess the effect of botulinum toxin type A (BTX-A) for the management of gummy smile and evaluate its stability after administrating BTX-A clinically and using electromyography.

**Methods.** The investigators designed and implemented a prospective clinical study on 10 patients with a gummy smile. Patients with different types of gummy smile were injected with BTX-A in the levator muscles of the upper lip and were followed for six months. The effect of BTX-A was evaluated clinically and using electromyography preoperatively and after two weeks and three and six months. Statistical analyses were carried out using repeated measures ANOVA and post hoc Bonferroni tests for pairwise comparisons.

**Results.** The sample consisted of 10 patients with an anterior gummy smile (n=3), posterior gummy smile (n=2), mixed gummy smile (n=3), and asymmetrical gummy smile (n=2). There were significant differences (*P* < 0.001) between the mean gingival display and compound muscle action potential at two-weeks and three-month follow-ups. The maximum result was obtained at the two-week interval. The mean gingival display and C-MAP values increased slightly at the three-month postoperative interval and gradually increased to the baseline values at six-month follow-up.

**Conclusion.** BTX-A is an effective, minimally invasive, and temporary treatment modality for gummy smiles. The electromyographic study is a convenient method for assessing changes in the upper lip muscle contractility to quantify the effect of BTX-A in the treatment of gummy smile.

## Introduction

According to Hulsey, “A smile is one of the most effective means by which people convey their emotions.”^1^ Just as a beautiful smile can serve as a powerful communication tool, an unpleasant smile can have an equally powerful negative impact. A gummy smile is a condition in which there is excessive exposure of the maxillary gingiva during a smile.^2^ It is an orofacial aesthetic disorder in which more than 3 mm of the maxillary gingiva is shown during normal smiling.^3^ It is further classified based on the area of excessive gum exposure as anterior, posterior, mixed, and asymmetrical gummy smile.^4^ Etiologic factors for the gummy smile can be skeletal, muscular, or gingival. Muscular cause of a gummy smile includes the upper lip’s hyperactive elevator muscles, pulling the upper lip way higher to expose excessive maxillary gingiva.

The muscles involved in smiling are the levator labii superioris alaeque nasi (LLSAN), levator labii superioris (LLS), zygomaticus major (ZM), zygomaticus minor (ZMn), depressor septi nasi, risorius, orbicularis oris, and levator anguli oris. Primarily, the hyperactivity of LLSAN, LLS, ZM, and ZMn are responsible for a gummy smile.^5^ Many surgical and non-surgical options have been described in the literature for the management of gummy smile, including Lefort I osteotomy, crown lengthening procedures, maxillary incisor intrusions, micro-implants, headgears, self-cured silicone implants injected at anterior nasal spine, myotomy, and partial resection of LLS with muscle re-positioning.^6^

The use of botulinum toxin in the treatment of gummy smile is relatively new. The neurotoxin prevents the release of acetylcholine at the neuromuscular junction, resulting in the relaxation of the upper lip’s elevator muscle.^7^ This causes the lip to lengthen, thus camouflaging the gummy smile. Polo^5^ first reported the use of botulinum toxin type A (BTX-A) for the treatment of gummy smile in 2005. Although multiple case reports and case series have been published on the application of BTX-A to manage gummy smile, there is a paucity of literature on clinical studies in this area.^4,5,8-10^ This present study was designed to evaluate the clinical effect of BTX-A to improve gummy smile. Electromyography was used to analyze the impact of BTX-A on the contraction ability of levator muscles of the upper lip for the first time.

In this study, patients with different types of gummy smile were injected with BTX-A and were followed up for six months. The study aimed to clinically assess the effectiveness of BTX-A in treating the gummy smile and study the stability of its outcomes. The effect of BTX-A in the treatment of gummy smile was also evaluated using electromyography. The compound muscle action potential of the levator group muscles was recorded preoperatively and after the injection procedure at two-week, three-month, and six-month intervals to assess changes in the muscles’ contraction ability.

## Methods

The investigators designed and implemented a prospective clinical study to assess the effect of BTX-A in the management of gummy smile and evaluate the stability of gummy smile correction after injecting BTX-A clinically and using electromyography. The present study was carried out in the Department of Oral and Maxillofacial Surgery, School of Dental Sciences, KIMSDU, Karad, after approval by the Institutional Ethical Committee (IEC/PG-D/101, dated; Nov 8, 2016). All the cases diagnosed with a gingival smile from November 2016 to August 2018 were considered for the study. Subjects eligible for the study had a gingival display of more than 3 mm, were 18‒35 years old, and were willing to participate in the study and follow-up visits. Subjects were excluded if they had gingival smile due to delayed passive eruption, had known allergy to albumin and/or botulinum toxin, were using medications, such as aminoglycosides, anti-cholinesterase, or other agents interfering with neuromuscular transmission, and were pregnant/lactating.

Ten patients were enrolled in the study after signing an informed written consent form. Of the 10 patients, the total number of females enrolled was seven, with three males. A detailed case history, including the patient’s general and systemic examination, was recorded. Based on the area of the gingival display, the patients were classified as having an anterior, posterior, mixed, or asymmetric gummy smile. Patients with a gingival display of > 3 mm between the maxillary canines were classified as anterior gummy smile, whereas those with a display of > 3 mm behind the maxillary canines were classified as having a posterior gummy smile. Patients with > 3 mm exposure between the maxillary canines and posterior to them were classified as having mixed gummy smile. Patients with gingival exposure of > 3 mm on either the right or left side only were classified as having an asymmetrical gummy smile. The parameters evaluated for this study were:

- Clinical measurement of gingival display
- Electromyography study (using C-MAP measurement)
- Patient satisfaction score

### Clinical measurement of gingival display

**Figure 1:**
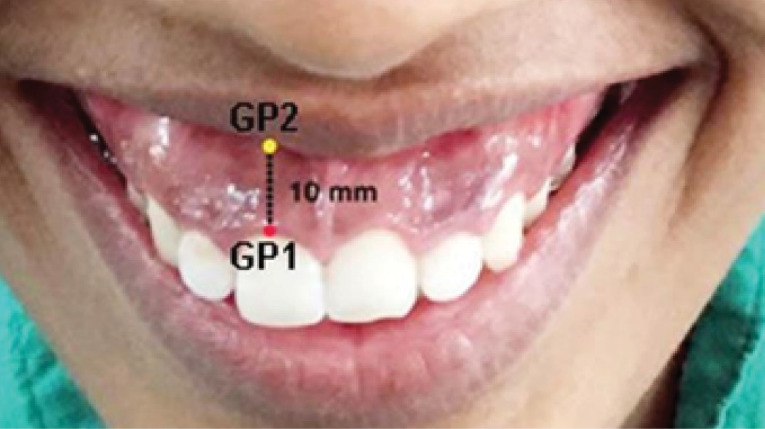
Measurement of the gingival display, defined as the distance between free gingival margin in the area of maximum gingival show (GP1) and lowest margin of the upper lip (GP2) on the photograph processing software.

Full-face front smiling photographs were taken using a digital camera (Canon EOS 1200D EF-S 18-55 IS II lens, intraoral shutter speed: 1/80 sec, focal length: f-16, ISO: 200). The photographs were captured standardized, non-posed, spontaneous smiles. The photographs were standardized by measuring the height and width of the crowns of the central incisors and premolars before and after injections. Measurements of gum exposure were only considered when the control measurements were the same in the pre- and post-injection photographs. All the photographs were then loaded in a laptop (Dell Inspiron Ultrabook 14z 5423 Ultrabook [Core i3 3rd Gen/4 GB/500 GB/Windows 8]) and processed using Adobe Photoshop software (version 7.0). Guiding points were marked, and measurements were recorded on the software. Guiding point-1 (GP1) was marked at the free gingival margin at the center of teeth where the gingival display was maximum on smiling, and Guiding point-2 (GP2) was marked over the inferior margin of the upper lip corresponding to GP1 (Figure 1). The measurements of total gingival display on smiling were recorded as the distance between GP1 and GP2. The measurements were made preoperatively and postoperatively during follow-up visits at two-week, three-month, and six-month intervals.

### Electromyography study (C-MAP measurement)

All the patients were subjected to an electromyographic study using C-MAP measurement to evaluate the electrical activity of the levator muscles of the upper lip. The compound muscle action potential is an electromyography investigation that measures the simultaneous action potentials for a group of muscle fibers in the same area. The C-MAP procedure included the use of surface electrodes applied over the group of muscles to be studied. These electrodes included ground, active, and inactive electrodes. The ground electrode provided insulation and was placed on the patient’s forehead. The active electrode (an active arm of the circuit) was placed on the muscle to be studied. The inactive electrode balanced the circuit and was placed over the chin region. The circuit was activated by stimulating the facial nerve at the tragus using a nerve stimulator. The C-MAP of levator muscles was recorded by muscle animation (by asking the patient to smile). The whole electrode assembly was attached to computer-aided software that recorded the compound muscle action potential. For patients with an anterior gummy smile, the active electrode was placed on the overlapping points of levator lip muscles lateral to the ala of the nose. In the patients with a posterior gummy smile, the active electrode was placed on zygomaticus muscles at the highest prominence of the cheek. For patients with a mixed gummy smile, C-MAP was recorded at both sites. In patients with an asymmetrical gummy smile, it was recorded over zygomaticus muscles on the affected side.

C-MAP was measured based on the mean amplitude calculated from peak to peak and plotted on a graph. It was measured by splitting the action potential into five subunits, and the mean value was recorded. C-MAP of patients with an anterior gummy smile was recorded at the right and left levator group of muscles bilaterally, and the mean amplitude was measured. Similarly, in patients with a posterior gummy smile, C-MAP of both right and left zygomaticus muscles was recorded, and the mean amplitude was measured. In patients with a mixed gummy smile, C-MAP of both the levator and zygomaticus muscles was recorded bilaterally, and the mean was calculated. C-MAP in patients with an asymmetrical gummy smile was recorded at the zygomaticus group of muscles on the affected and unaffected side, and the mean was calculated. The procedure was carried out preoperatively and repeated during the follow-up visits at two-week, three-month, and six-month intervals. Depending on the decrement of C-MAP amplitude, the quantitative extent of the neuromuscular blockade was assessed.

### Patient satisfaction score

Patient satisfaction score was measured by a questionnaire submitted to all the patients at the end of the six-month follow-up. The questionnaire consisted of queries regarding patient satisfaction, any discomfort during the procedure, and recommendation to oneself and others to undergo the same treatment.

### Botulinum injection procedure

**Figure 2:**
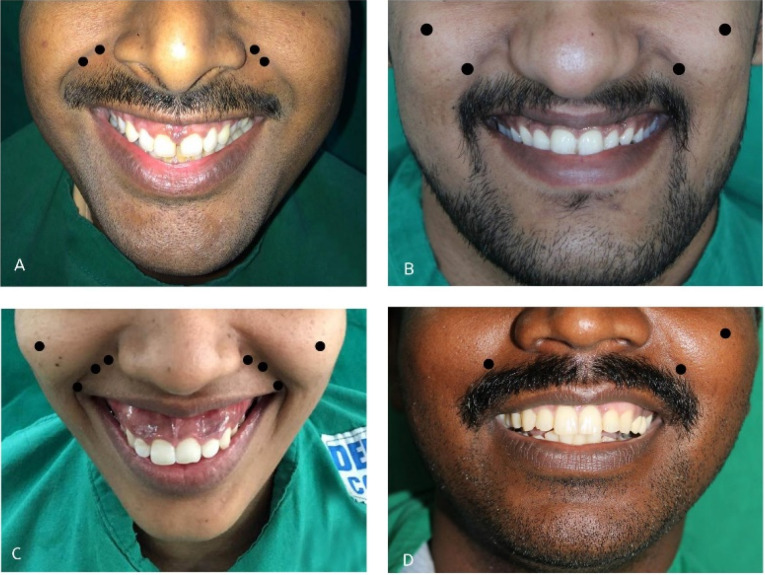
Injection points for anterior (A), posterior (B), mixed (C), and asymmetric (D) gummy smile.

Following the preoperative assessment (the clinical measurement of gingival exposure and electromyography study), the patients were prepared for the treatment. The botulinum toxin used in this study was botulinum toxin type A (SIAX, Aakar Pharmaceuticals, India). The BTX-A was provided as a freeze-dried powder and reconstituted by diluting it with 0.9% normal saline, according to the manufacturer’s recommendation; 8 mL of normal saline was added to 100 units (U) of vacuum-dried powder to yield 1.25 U of botulinum toxin per 0.1 mL. The groups of muscles to be injected were palpated by asking the patient to smile spontaneously. For the anterior gummy smile, the muscles injected were LLSAN and LLS. The landmark for injection of LLSAN was at a point 3-5 mm lateral to the ala of the nose, whereas, for LLS, the point of injection was at the junction of upper 1/3 and lower 2/3 of the nasolabial fold (Figure 2A). For the posterior gummy smile, the muscles injected were ZM and ZMn group of muscles at its origin and insertion. The landmark for injection of ZM-ZMn origin was at the point of the prominence of cheek and at the insertion point, which was the most lateral portion of the nasolabial fold while smiling (Figure 2B). Patients with a mixed gummy smile were injected in both the levator and zygomaticus group of muscles (Figure 2C). Patients with an asymmetrical gummy smile received BTX-A at the origin and insertion points of the ZM and ZMn group of muscles on the affected side and only at the insertion point of ZM and ZMn on the unaffected side (Figure 2D). 1.25 U of BTX-A was injected per site. Patients were discharged on the same day and were advised to avoid sun exposure and vigorous rubbing of the face. All the patients were instructed to take antihistaminic medications (Tab. Avil 25 mg) in case of an allergic reaction and advised to report back to the department in case of any adverse reaction. All the patients were informed about the re-injection procedure if any further correction was needed. Only one patient with a mixed gummy smile and gingival display of 10 mm was injected with an additional 1.25 U at the two-week follow-up.

The patients were recalled at the two-week, three-month, and six-month intervals. Peri-oral photographs were taken to analyze the changes in the values of guiding points, and C-MAP measurements were repeated to evaluate the decrement in the electrical activity of the levator muscles of the upper lip. All the data were compiled and tabulated to assess the results of the study.

## Results

Statistical analyses were performed after data collection. Statistical tests were carried out to compare gingival display and compound muscle action potential measurements preoperatively and at two-week, three-month, and six-month postoperative intervals. The descriptive statistics measured for gingival display (in mm) and C-MAP (in µv) (Table 1) were expressed as means and standard deviations. The gingival display and C-MAP in all the patients for six months were compared by repeated-measures ANOVA and post hoc Bonferroni tests for pairwise comparisons. The mean reduction of the gingival display from the preoperative period (6.20±1.751) to the two-week postoperative interval (3.30±0.949) was 2.900, which was statistically significant (with P < 0.001) by both ANOVA and post hoc Bonferroni test (Table 2). The mean gingival display at three-month follow-up was 4.40±1.174 mm, almost returning to the baseline value at six-month follow-up (5.60±1.506 mm). The mean C-MAP (in µv) preoperatively was 254.70 (±236.66), which decreased to 114.80 (±94.11) at the two-week follow-up. The mean C-MAP value increased gradually, measuring 145.10 (±115.73) at the three-month follow-up and 229.45 (±204.65) at the six-month follow-up. Although there was a noticeable decrease in the mean C-MAP during the follow-up, the difference was statistically significant only at the two-week and three-month postoperative intervals by Bonferroni test (*P* = 0.015) (Table 3).

**Table 1:** Descriptive statistics for gingival display (in mm) and C-MAP (in microvolts) over time

	**Gingival display (mm)**				**C-MAP values (µV)**			
	Min	Max	Mean	SD	Min	Max	Mean	SD
Pre. OP	4	10	6.20	1.751	79.00	794.00	254.7000	236.66270
PO. 2 weeks	2	5	3.30	0.949	31.50	320.50	114.8000	94.11701
PO. 3 months	3	6	4.40	1.174	48.00	400.00	145.1000	115.73384
PO. 6 months	3	8	5.60	1.506	79.00	670.00	229.4500	204.65180

Min = minimum, Max = maximum, SD = standard deviation, Pre OP = preoperative, PO = postoperative.

**Table 2:** Pairwise comparison of mean gingival display (in mm) between four time intervals by Bonferroni test

Gingival display (Period of Interval)		Significance	95% Confidence interval for difference	
			**Lower bound**	**Upper bound**
Pre op	Post op 2 weeks	< 0.001*	1.531	4.269
Pre op	Post op 3 months	0.004*	0.592	3.008
Pre op	Post op 6 months	0.143	-.144	1.344
Post op 2 weeks	Post op 3 months	0.001*	-1.704	-.496
Post op 2 weeks	Post op 6 months	< 0.001*	-3.309	-1.291
Post op 3 months	Post op 6 months	0.006*	-2.039	-.361

*The mean difference is significant at the 0.05 level

**Table 3:** Pairwise comparison of compound muscle action potential values (in microvolt) among period of four time intervals by Bonferroni test

Compound muscle action potential		Significance	95% confidence interval for difference	
			**Lower bound**	**Upper bound**
Pre op	Post op 2 weeks	0.080	-13.538	293.338
Pre op	Post op 3 months	0.121	-21.307	240.507
Pre op	Post op 6 months	0.343	-13.721	64.221
Post op 2 weeks	Post op 3 months	0.015*	-54.865	-5.735
Post op 2 weeks	Post op 6 months	0.063	-234.591	5.291
Post op 3 months	Post op 6 months	0.106	-182.306	13.606

*The mean difference is significant at the 0.05 level.

**Figure 3:**
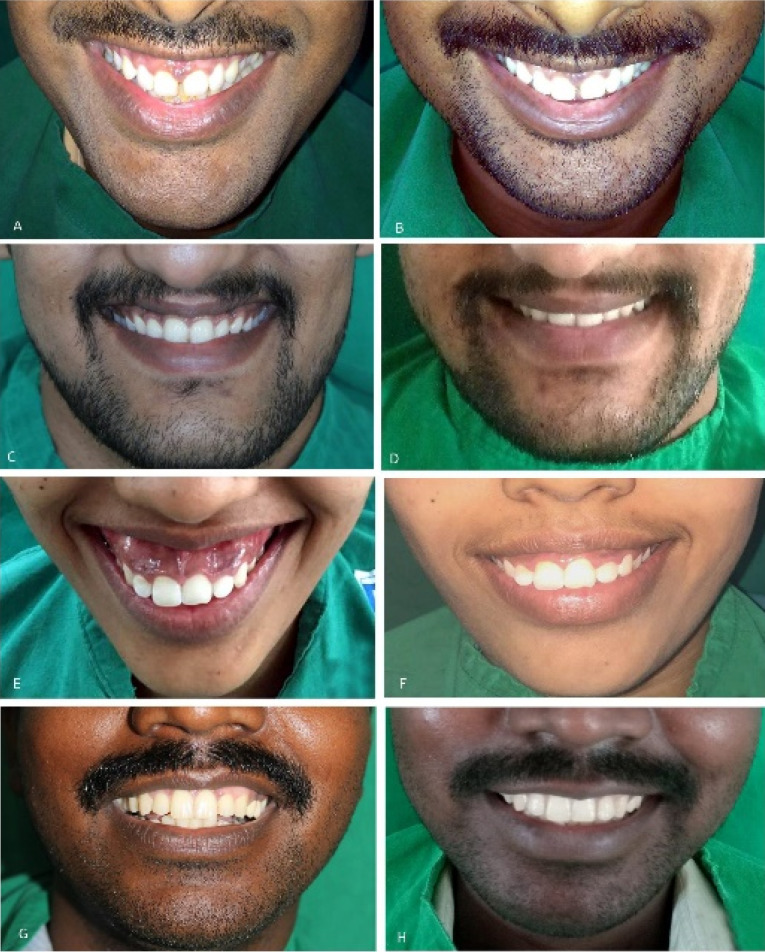
Patients with gummy smile treated with BTX-A. Anterior gummy smile with a gingival display of 5 mm (A); the postoperative result (B); posterior gummy smile with a gingival display of 5 mm (C); the postoperative result (D); mixed gummy smile with an anterior gingival display of 10 mm and posterior gingival display of 6 mm (E); the postoperative result (F), Asymmetric gummy smile with gingival display of 5mm (G), postoperative result (H).

The patient satisfaction scores were analyzed based on the questionnaire administered to patients at the six-month follow-up. When asked about the treatment satisfaction, 30% of the patients were satisfied, while 70% were fully satisfied with the treatment; 50% did not feel any discomfort during the injection procedure, while 50% felt a slight pain on injection. 70% of the patients agreed to undergo the treatment again in the future. When asked about their recommendation of the BTX-A treatment to other family members, 60% strongly agreed with the recommendation. All the patients were satisfied with the treatment and did not require a second except for one patient with a gingival display of 10 mm, who desired further correction. Figure 3 presents the clinical results of the treatment of various types of gummy smile managed with BTX-A. None of the patients had any adverse reactions to BTX-A injections while undergoing treatment or during the follow-up visits.

## Discussion

Smile is an essential component of an individual’s appearance and expresses a sense of happiness, success, affection, courtesy, confidence, and kindness. A balanced smile is created by the harmony between three components: upper lip (levator) muscles, maxillary anterior teeth, and anterior maxillary skeleton. A discrepancy of any one of the components can result in an unpleasant smile. A gingival smile is defined as a smile where more than 3 mm of maxillary gingiva is exposed when a person smiles.

Multiple etiological factors are attributed to the occurrence of gummy smile, which can be widely classified as skeletal, dental, muscular, or a combination of these. The choice of treatment for gingival smile depends upon the underlying cause. Increased gingival display due to excessive vertical growth of the maxilla is ideally treated by maxillary osteotomy procedure. Gummy smile due to dental etiology can be attributed to a delayed passive eruption, abnormal size of maxillary incisors, or gingival hypertrophy/hyperplasia.^10^ Muscle hyperactivity is the second common cause of gummy smile after vertical maxillary excess.^11^ Traditionally, such gingival smiles have been treated by various surgical procedures, including myotomy and myectomy of levator muscles of the upper lip.^4^

Management of gummy smile using botulinum toxin is a relatively non-invasive modality and was reported first by Polo^5^ in a pilot study in 2005. However, there is limited evidence in the literature for the use of BTX-A to manage gummy smile. A literature search for the use of BTX-A in the management of gummy smile in PubMed, Embase, Scopus, and Web of Science databases yielded 33 articles,^12^ 10 of which were prospective clinical studies.^1,2,4-6,8-11,13^ No randomized controlled trials (RCT) were identified.

The present study was designed to evaluate the effect of BTX-A in the management of gummy smile, both clinically and using electromyography. Although the application of electromyography guided injection was mentioned by Polo,^5^ its use to analyze the effect of BTX-A on the contraction ability of levator muscles of the upper lip is reported for the first time through this study.

With very limited evidence available in the literature, there is a lack of a defined protocol for the use of BTX-A for the management of gummy smile. The dose, site, and types of gummy smile treated are different between the reported clinical studies. In the present study, the use of BTX-A was based on the type of gummy smile, which is similar to the protocol reported by Mazzuco et al.^4^

The choice of the site of injection is different between different studies. The majority of authors used the overlapping points of LLSAN and LLS, LLS, and ZMn as the landmark for BTX-A injections,^2,5,6,10,11^ while few studies suggested injections into the LLSAN and LLS muscles.^1,9,13-15^ Hwang et al^13^ assessed the morphological characteristics of upper lip musculature. The authors identified a common point of intersection of LLSAN, LLS, and ZMn for injecting BTX-A and named it Yonsei’s point. This landmark is identified 1 cm lateral to the ala of the nose and 3 cm from the corner of the mouth. Sucupira andAbramovitz^8^ suggested only one point of injection at the LLSAN, whileMazzuco et al^4^ suggested injection points at the LLSAN, LLS, ZM, and ZMn muscles.In the present study, the site of injection was determined by the type of gummy smile. As the levator muscles (LLSAN and LLS) of the upper lip are responsible for lifting the central portion of the lip superiorly, patients with an anterior gummy smile were injected at LLSAN and LLS.

Similarly, hyperactivity of the zygomaticus group of muscles (ZM and ZMn) are responsible for lifting the upper lip laterally, resulting in a posterior gummy smile. Patients with a posterior gummy smile received an injection at the origin and insertion areas of ZM and ZMn. Patients with mixed gummy smile were injected at all four muscles. In contrast, patients with an asymmetrical gummy smile received injections at the origin and insertion points of ZM and ZMn on the affected side. The unaffected side was injected at the insertion of ZM and ZMn to balance the smile.

Botulinum toxin used in the present study was BTX-A (Siax, by Aakar Medical Technologies Pvt. Ltd.). It is commercially available as a freeze-dried powder, which was activated by diluting it in 8 mL of normal saline to yield 12.5 U per ml. The shelf-life of freeze-dried botulinum toxin is 24 months, and it is stored in the refrigerator at 2‒4ºC. Botulinum toxin was used within 4 hours of its reconstitution. However, the literature shows that botulinum toxin maintains its potency for a more extended period and can be safely used within one month of reconstitution.^16^

Like the site(s) of injection, the reported doses of BTX-A used to manage gingival smile are different. Polo^5^ advised that the dose and injection sites of botulinum toxin should be tailored to the severity of gingival display. One injection site is recommended when the gum exposure is < 7 mm, and two injection sites when it exceeds 7 mm. Doses of injected BTX per side reported in the literature range from 2 U to 7.5 U per side.^1,2,4-6,8-11^ In the present study, a dose of 1.25 U was used per injection site. A lower dose was chosen to avoid any possibility of overcorrection, resulting in an unaesthetic smile. All the patients were informed of the need for re-assessment on follow-up, and if further correction was desired, an additional dose was given. Of the 10 patients treated, only one (with a mixed gingival display of 10 mm) required an extra dose for further correction.

The effect of BTX-A starts after 1‒3 days^10^ and reaches its maximum by two weeks,^10^ gradually decreasing in six months.^5^ There are individual variations in the duration of action, depending on the dose, number of muscles injected, previous injection, and patient age.^17^ The duration of the effect of BTX-A is longer in patients receiving repeated doses. This is said to happen because the repeated dose causes partial loss of muscle contractibility, leading to partial muscle atrophy.^12^ Patient’s age is another factor, and as age advances, tissue laxity develops, prolonging the duration of action of BTX-A. In the present study, the maximum effect was seen at the two-week postoperative interval, which gradually returned to almost the baseline condition at the end of six months. The difference in the mean gingival display from the preoperative period (6.20±1.751) to the two-week postoperative interval (3.30±0.949) was 2.900, which was statistically significant (*P* < 0.001). The mean gingival display at the three-month follow-up was 4.40±1.174 mm, which almost returned to the baseline value at the six-month follow-up (5.60±1.506 mm).

In addition to the clinical evaluation, electromyography (C-MAP) was used to study the change in contraction ability of the levator muscles of the upper lip in response to BTX-A injection. C-MAP represents the sum of muscle action potentials of a group of muscle fibers. C-MAP values for the patients reduced, and the mean amplitude of C-MAP decreased significantly after two weeks (114.8 µv) after BTX-A injection. The C-MAP increased slightly at the three-month follow-up (145.1 µv) and nearly returned to the baseline value after six months (229.4 µv). The results were statistically significant at the two-weeks and three-month postoperative intervals by Bonferroni’s test (*P* = 0.015). Based on clinical assessment and electromyography readings, it was observed that BTX-A was effective in the management of gummy smile, and the results persisted up to three months and then gradually returned to baseline after six months. Although the effect was transitory, the patients were fully satisfied with the treatment and willing to undergo the procedure again.

Although several undesirable effects of treatment of gummy smile with botulinum toxin have been reported, most of them have been minor, including an asymmetrical smile,^2^ thecollapse of the oral commissure resulting in a sad appearance, lengthening of the upper lip,^4^ joker smile,^6^ inferior lip protrusion, drooling, and difficulty in smiling, speaking, or eating.^4^ In the present study, no adverse effects were seen in any of the patients.

## Conclusion

The management of gummy smile using BTX-A is a transient method. It is a relatively safe, simple, and minimally invasive treatment modality. The patients in the present study were selected carefully, and the site of injection depended on the type of gingival display. C-MAP measurement introduced in this study is a useful adjunct to studying the postoperative stability of the results along with clinical evaluation.

## Authors’ contributions

PPM: Conduction of the clinical study, Drafting of the initial manuscript. KN and AP: Conduction of the clinical study, corrections of the prepared draft of manuscript. AGJ: Guidance for the electromyographic study of the subjects enrolled for the study.

## Acknowledgements

The authors of the study would like to acknowledge all the patients who agreed to participate in the study. Also, the statistician for providing the authors with elaborate and simplified analysis of the study results.

## Funding

None.

## Competing Interests

None.

## Ethics Approval

Krishna Institute of Medical Sciences, Deemed to be University, IEC/PG-D/101, dated; 8^th^ November 2016.
